# Redox Regulation of Insulin Degradation by Insulin-Degrading Enzyme

**DOI:** 10.1371/journal.pone.0018138

**Published:** 2011-03-23

**Authors:** Crystal M. Cordes, Robert G. Bennett, Gerri L. Siford, Frederick G. Hamel

**Affiliations:** 1 Research Service, Omaha Veterans Affairs Medical Center, Omaha, Nebraska, United States of America; 2 Department of Internal Medicine, University of Nebraska Medical Center, Omaha, Nebraska, United States of America; 3 Department of Pharmacology and Experimental Neuroscience, University of Nebraska Medical Center, Omaha, Nebraska, United States of America; 4 Department of Biochemistry and Molecular Biology, University of Nebraska Medical Center, Omaha, Nebraska, United States of America; University of Bremen, Germany

## Abstract

Insulin-degrading enzyme (IDE) is a thiol sensitive peptidase that degrades insulin and amyloid β, and has been linked to type 2 diabetes mellitus and Alzheimer's disease. We examined the thiol sensitivity of IDE using S-nitrosoglutathione, reduced glutathione, and oxidized glutathione to distinguish the effects of nitric oxide from that of the redox state. The in vitro activity of IDE was studied using either partially purified cytosolic enzyme from male Sprague-Dawley rats, or purified rat recombinant enzyme. We confirm that nitric oxide inhibits the degrading activity of IDE, and that it affects proteasome activity through this interaction with IDE, but does not affect the proteasome directly. Oxidized glutathione inhibits IDE through glutathionylation, which was reversible by dithiothreitol but not by ascorbic acid. Reduced glutathione had no effect on IDE, but reacted with partially degraded insulin to disrupt its disulfide bonds and accelerate its breakdown to trichloroacetic acid soluble fragments. Our results demonstrate the sensitivity of insulin degradation by IDE to the redox environment and suggest another mechanism by which the cell's oxidation state may contribute to the development of, and the link between, type 2 diabetes and Alzheimer's disease.

## Introduction

Insulin-degrading enzyme (IDE, EC 3.4.24.56) hydrolyzes a number of small (<12,000 MW) peptides, notably insulin and Alzheimer's amyloid β peptide. As such, it has been implicated in the etiology of both type 2 diabetes (T2DM) and Alzheimer's disease (AD). The Goto-Kakizaki rat, an animal model of T2DM, contains mutations in the IDE gene and exhibits elevated blood glucose and insulin levels [1]. IDE knockout mice are both glucose-intolerant and hyperinsulinemic, supporting the concept that IDE is important in the maintenance of normal blood glucose and insulin levels [2]. Human genetic studies have linked polymorphisms in the IDE gene to an increased risk for insulin resistance and T2DM [3], [4], [5]. IDE hypofunction has been shown to contribute to the accumulation of Aβ plaques in animal models of AD [2]. Human genetic studies have also linked IDE to AD [6], [7], [8]. Thus, factors that affect the activity of IDE, such as post-translational modification, could have significant impact on the progression of these diseases.

IDE is a ubiquitously expressed zinc metalopeptidase that is inhibited by thiol reactive agents [9]. The cysteines most likely responsible for this thiol sensitivity have been identified [10]. We and others have shown that nitric oxide (NO) is capable of reacting with IDE and inhibiting activity [11], [12]. Both T2DM and AD have been associated with a chronic inflammatory state, which may result in locally increased iNOS expression and NO release [13], [14], [15], [16], [17]. In further study of this phenomenon, we have used the NO donor S-nitrosoglutathione (GSNO), which may be a physiological sink for NO in cells [18], [19]. Our results further characterize our previous report, but also demonstrate a role for glutathione in the control of insulin degradation by interaction with both IDE and partially degraded insulin itself. This is significant because of the change in cellular redox state found in both diabetes and AD.

In this study, we examined the effects of S-nitrosoglutathione (GSNO), reduced glutathione (GSH), and oxidized glutathione (GSSG) on IDE function. We show that GSNO inhibits IDE-mediated degradation of two IDE substrates, insulin and Aβ, and that IDE-mediated regulation of the proteasome is inhibited. We also found that GSSG inhibits IDE while GSH increased the breakdown of partially-degraded insulin. Taken together, these findings demonstrate potential pathways by which imbalances in the oxidative state may contribute to the pathology of T2DM and AD, and represent potential therapeutic targets for the treatment of these diseases.

## Materials and Methods

### Chemicals

S-nitrosoglutathione (GSNO), GSSG, GSH, and the fluorogenic proteasome substrate succinyl-leu-leu-val-tyr-7-amido-4-methyl coumarin (LLVY) were purchased from Sigma (St. Louis). GSNO(ox) is GSNO that has been allowed to decompose to release NO from the compound, which can be followed by measuring absorbance at 340 nm [20], [21]. Decomposition was accomplished by incubating GSNO at room temperature in the dark for 72 hours. ^125^I-human recombinant insulin and ^125^I-β-amyloid were purchased from GE Healthcare Life Sciences (Piscataway) and Phoenix Pharmaceuticals (Burlingame), respectively. Polyclonal anti-IDE antibody and the monoclonal anti-glutathione antibody were purchased from Millipore. All other chemicals were of at least reagent grade. *S*-methyl methanethiosulfonate (MMT) was from Aldrich (St. Louis). *N*-[6-(Biotinamido)hexyl]−3′−(2′-pyridyldithio) propionamide (biotin-HPDP) was from Thermo Scientific (Rockford). IRDye 800 CW® Streptavidin was from LiCor Biosciences (Lincoln).

### Enzyme Preparation

Male Sprague-Dawley rats were maintained and used in accordance with the National Institutes of Health Guide for the Care and Use of Laboratory Animals, under a protocol (ID# 00319) approved by the Subcommittee of Animal Studies and the Research and Development Committee of the Omaha Veterans Affairs Medical Center. Insulin-degrading enzyme/proteasome was prepared from rat muscle or liver by ultracentrifugation and ammonium sulfate precipitation, similar to that described previously [22]. This preparation was used, rather than a more purified form of IDE, so that we could study the interaction with the proteasome. IDE is the only insulin-degrading activity in this preparation (data not shown). Purified proteasome was obtained from Sigma (St. Louis, MO). Purified IDE was purchased from EMD Chemicals, Inc (Gibbstown, NJ).

### Insulin Degradation

The degradation of ^125^I-insulin and ^125^I-β-amyloid was measured by the trichloroacetic acid (TCA) solubility method. GSSG, GSH, and GSNO, and oxidized GSNO (GSNO(ox)) were solubilized in the assay diluent (100 mM Tris-Cl). An aliquot of rat enzyme was pretreated with GSNO and GSNO(ox) for 1 hour prior to the insulin degradation assay. The enzyme preparation was incubated with increasing concentrations of GSH, GSSG, GSNO, and GSNO(ox) (with and without ascorbate [10^−3^ M]) and ^125^I-insulin or ^125^I-β-amyloid for 15 minutes at 37°C. The reaction was stopped by addition of 0.5% (final) BSA and 10% (final) TCA. Samples were centrifuged, and the supernatant and pellet counted using a gamma counter, with data expressed as percent degradation relative to untreated enzyme. Substrate incubated without enzyme was subtracted as background solubility. For the enzyme inactivation experiments, prior to the addition of BSA and TCA, the samples were heated for 8 minutes at 90°C, cooled to approximately 40°C, 10^−2^ M GSH was added, and the samples were incubated an additional 15 minutes at 37°C before the reaction was stopped.

### Nitrosylation of IDE

Nitrosylation of IDE was assessed by a modification of the biotin switch method [23], [24]. The samples were acetone precipitated between each step. Purified IDE was incubated without and with 10^−4^ M GSNO and GSNO(ox) for 2 hours at 37°C in 100 mM Tris, pH 7.5. The samples were resuspended in HENS buffer (0.25 M HEPES, 1 mM EDTA, 0.1 M neocuprione, 1% SDS) with 20 mM MMT and incubated for 30 minutes at 50°C to block free sulfhydryls. The precipitated samples were resuspended in HENS buffer without or with 2.6 mM biotin-HPDP and ascorbate, and incubated for 1 hour at room temperature. The samples were run on non-reducing gels, and transferred to PVDF membrane and probed with IRdye streptavidin. Blots were visualized on an Odyssey® infrared imaging system.

### Proteasome Activity

The degradation of the fluorogenic peptide substrate, LLVY, was used as a measure of the chymotrypsin-like activity of the proteasome [25]. Aliquots of rat muscle enzyme preparation or purified proteasome were incubated with increasing concentrations of GSH, GSSG, GSNO and GSNO(ox) with and without ascorbate [1x10^−3^ M] in a 96-well black plate at 37°C for 1 hour. LLVY was added (13 µM final concentration), and florescence measurements (excitation/emission: 355/460 nm) were taken at 30, 60, 90, and 120 minutes post-addition using a Perkin-Elmer Victor^3^ 1420 multilabel counter. Data are expressed as the rate of change in fluorescence units and normalized to the activity of untreated enzyme.

### HPLC Analysis of Insulin Degradation Products

Rat liver IDE was preincubated with GSNO or GSNO(ox) for 1 hour at 37°C in 100 mM Tris-Cl buffer. For experiments with GSH or GSSG, the enzyme was not pretreated. The enzyme samples were then incubated with approximately 5×10^5^ cpm ^125^I-insulin for 5 minutes at 37°C, and then applied directly to a reversed-phase HPLC column, similar to that previously described [26]. Fractions of the eluate were collected and analyzed using a gamma-counter to determine the elution profile of radioactivity.

### Glutathionylation of insulin-degrading enzyme

Partially purified rat enzyme was incubated with GSH, GSSG, GSNO, or GSNO(ox) for 1 hour at 37°C. The samples were then mixed with loading dye without β-mercaptoethanol and resolved on polyacrylamide gels with SDS. Samples were then transferred to PVDF membranes and probed for IDE and glutathione modification.

### Data Analysis

Results are expressed as mean ± SEM. Data were analyzed using the Prism GraphPad statistical software (Version 5, La Jolla, CA). Significance was determined by one-way ANOVA with Dunnett's multiple comparison test.

## Results

**Figure 1:**
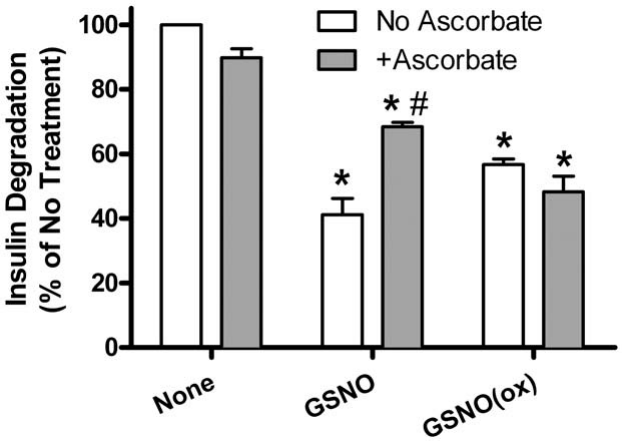
GSNO and GSNO(ox) inhibit IDE. The effect of 10^−4^ M GSNO or GSNO(ox) on ^125^I-Insulin degradation by partially purified liver IDE is shown in the absence (open bars) or presence (grey bars) of ascorbate [10^−3^ M]. Inhibition by GSNO (NO donor) is prevented by ascorbate, while GSNO(ox) inhibition is not. All graphs represent the mean ± SEM of at least three independent experiments. *P<0.01compared to no addition. #P<0.01 compared to GSNO without ascorbate.

**Figure 2:**
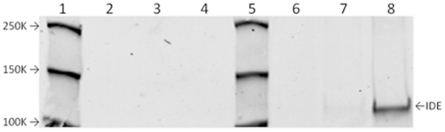
Nitrosylation of IDE by GSNO. The nitrosylation of purified recombinant IDE by GSNO, but not GSNO(ox) is demonstrated using the biotin switch method. IDE was either not treated (lanes 2 and 6), treated with 10^−4^ M GSNO(ox) (lanes 3 and 7) or 10^−4^ M GSNO (lanes 4 and 8). Lanes 2 through 4 show controls without the biotin reagent. Lanes 6 through 8 show enzyme subjected to biotin labeling. Lanes 1 and 5 are molecular weight markers with indicated MW. IDE at approximately 110 K MW is indicated.

The effects of the NO donor compound, GSNO and the control compound GSNO(ox) on the insulin-degrading activity of IDE are shown in Figure 1. Both compounds inhibit insulin degradation. The addition of ascorbate (10^−3^ M) partially reverses the inhibitory effect of GSNO, but had no effect on GSNO(ox). Ascorbate alone had no effect on insulin degradation. Since ascorbate is known to reverse potential nitrosylative effects, this suggests GSNO is working via NO donation. The nitrosylation of IDE by GSNO, but not GSNO(ox) is confirmed in Figure 2, using the biotin switch method. Neither GSNO nor GSNO(ox) had any qualitative effect on the insulin fragments generated as assessed by HPLC (data not shown), suggesting that nitrosylation decreases the rate of hydrolysis, but not the specificity of IDE.

**Figure 3:**
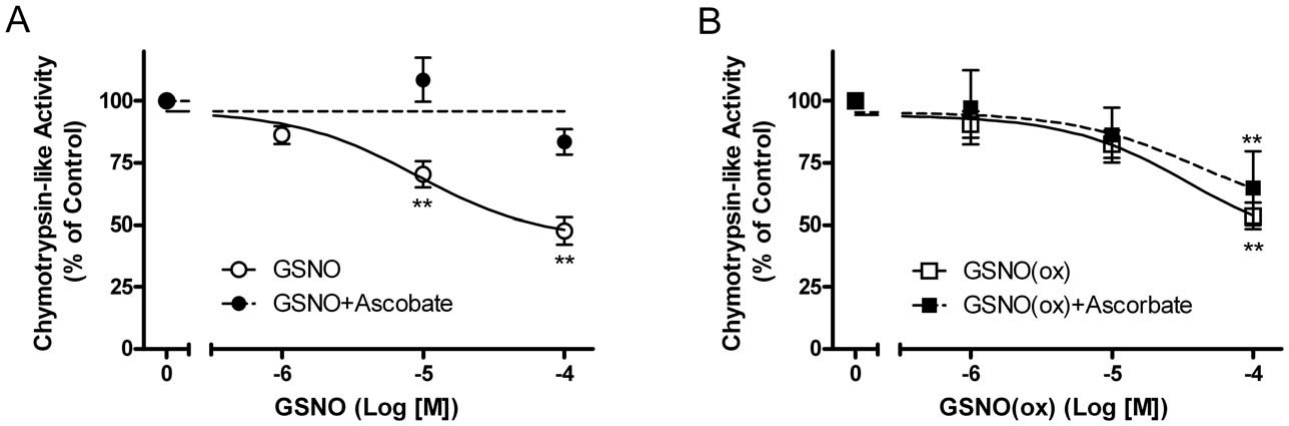
Proteasome chymotrypsin-like activity with GSNO and GSNO(ox) treatment. Partially-purified rat IDE enzyme with proteasome was treated with increasing concentrations of NO donor and assessed for chymotrypsin-like activity using a fluorogenic proteasome substrate. (A) Proteasome activity with GSNO (○), GSNO+Ascorbate [10^−3^ M] (•). (B) Proteasome activity with GSNO(ox) (□), GSNO(ox)+Ascorbate [10–3 M] (▪). Inhibition by GSNO (NO donor) is prevented by ascorbate, while GSNO(ox) inhibition is not. Mean ± SEM of at least three independent experiments; **P<0.01 compared to no addition.

**Figure 4:**
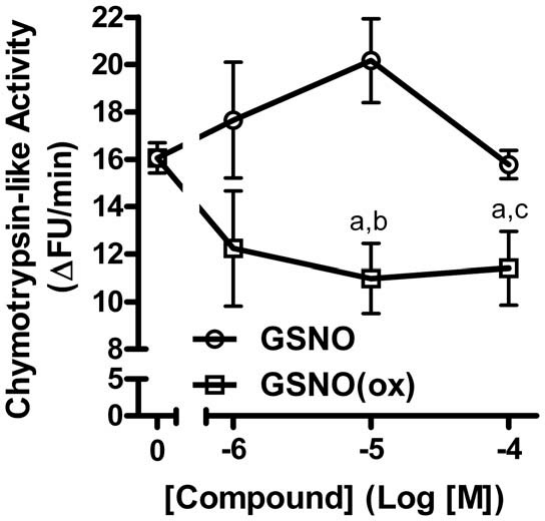
Effect of GSNO or GSNO(ox) on purified proteasome. Purified proteasome (no IDE) was treated with increasing concentrations of NO donor and assessed for chymotrypsin-like activity using a fluorogenic proteasome substrate; GSNO (○), GSNO(ox) (□). Purified proteasome is not susceptible to inhibition by NO. Mean ± SEM of three experiments; a is P<0.05 compared to no addition; b is P<0.05 GSNO(ox) compared to GSNO at 10^−5^ M; c is P = 0.058 GSNO(ox) compared to GSNO at 10^−4^ M.

Our lab has previously shown that substrates and inhibitors of IDE will decrease the chymotrypsin- and trypsin-like activities of the proteasome when it is isolated in a complex with IDE [27], [28]. Thus, we tested our *in vitro* rat enzyme preparation containing isolated IDE and proteasome complex to see whether or not GSNO or GSNO(ox) would have an effect on proteasome activity. We treated the IDE-proteasome complex with GSNO (Figure 3A) and GSNO(ox) (Figure 3B) in the presence and absence of ascorbate and examined the chymotrypsin-like activity. GSNO inhibited the proteasome activity by 50%, whereas in the presence of ascorbate the effect was greatly diminished. This indicates that the inhibitory effect observed with GSNO treatment of the proteasome is reversible, which was also seen in the insulin degradation assays. In contrast, GSNO(ox) inhibited proteasome activity, but was not reversed by ascorbate. However, the effect on the proteasome activity may have been due to direct action on the proteasome and not through IDE. Thus, we used a purified 20 S proteasome preparation to examine the effects of GSNO and GSNO(ox) on the chymotrypsin-like activity. GSNO had no effect on the proteasome, while GSNO(ox) did directly inhibit (Figure 4). Together with Figure 3, these results indicate NO does not affect the proteasome directly, but works though its reversible interaction with IDE. However, GSNO(ox) works on both enzymes and is not affected by ascorbate.

**Figure 5:**
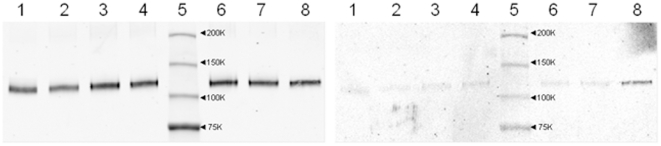
Glutathionylation of IDE by GSNO(ox). Post-translational modification of IDE by glutathionylation was measured by Western blotting with an anti-glutathione antibody (right panel). Anti-IDE blot of the same gel is shown in the left panel. Partially-purified IDE was left untreated (lane 1) or treated with GSNO [10^−6^, 10^−5^, or 10^−4^ M, lanes 2 to 4 respectively] or GSNO(ox) [10^−6^, 10^−5^, or 10^−4^ M, lanes 6–8, respectively]. Molecular weight markers, with their sizes indicated, are in lane 5. Lane 8 (right panel) shows increased glutathione staining with GSNO(ox), while GSNO had no effect. Blot is representative of 4 similar experiments.

Low-molecular weight nitrosothiols, such as GSNO, have been shown to glutathionylate protein cysteine residues, probably through reactive intermediates generated in its breakdown [29]. Thus, glutathionylation of IDE with GSNO and GSNO(ox) treatment was explored using an anti-glutathione antibody (Figure 5). The partially purified IDE preparation appears to show a low level of endogenous glutathionylation, which increases significantly with GSNO(ox) at 10^−4^ M. GSNO has been reported to decompose to the reduced and oxidized forms of glutathione, as well as oxidized glutathione derivatives [30], [31], suggesting that GSH or GSSG may modify IDE.

**Figure 6:**
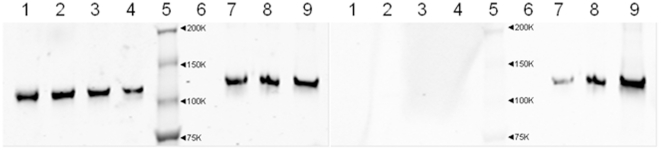
Glutathionylation of purified IDE by GSSG. Post-translational modification of IDE by glutathionylation was measured by Western blotting with an anti-glutathione antibody (right panel). Anti-IDE blot is shown on the left. Partially-purified IDE was left untreated (lane 1) or treated with GSH [10^−4^, 10^−3^, or 10^−2^ M, lanes 2 to 4 respectively] or GSSG [10^−4^, 10^−3^, or 10^−2^ M, lanes 7 to 9, respectively]. Molecular weight markers, with their sizes indicated, are in lane 5. Lane 6 is a blank. GSSG dose-dependently increased glutathionylation of IDE, while GSH had no effect. Blot is representative of 4 similar experiments.

However, there is conflicting evidence as to which component of GSNO decomposition is most effective at glutathionylation [31], [32], [33]. Therefore we examined the ability of both GSH and GSSG to glutathionylate IDE (Figure 6). The recombinant purified form of IDE showed no glutathionylation. GSH had no effect, whereas GSSG showed a dose-dependent increase in glutathionylation of IDE. Thus, inhibition by GSNO(ox) likely works by modification of IDE via GSSG. These findings prompted us to examine the effect of two potential GSNO decomposition products, GSH and GSSG, on IDE-mediated degradation of insulin and Aβ.

**Figure 7:**
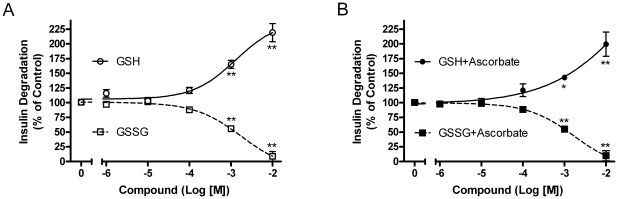
Effect of GSH and GSSG on insulin degradation. ^125^I-Insulin degradation by IDE is shown after treatment with GSH and GSSG in the presence and absence of ascorbate [10^−3^ M]. (A) Insulin degradation curve fit with increasing concentrations of GSH (○) and GSSG (□). (B) Insulin degradation curve fit with GSH+Ascorbate [10^−3^ M] (•) and GSSG+Ascorbate [10^−3^ M] (▪). GSSG inhibits IDE, while GSH appears to increase activity. Mean ± SEM of at least 3 independent experiments; *P<0.05, **P<0.01 compared to no addition.

The effect of GSH and GSSG on IDE-mediated degradation of insulin is shown in Figure 7. Partially purified rat IDE activity was significantly inhibited by at least 40% at 10^−3^ M of GSSG and by more than 90% at 10^−2^ M. Conversely, GSH appeared to have significantly *increased* insulin degradation, more than doubling TCA solubility at 10^−2^ M. A similar increase in solubility is seen with the reducing agent dithiothreitol (DTT, data not shown). Figure 7B shows the effect of GSH and GSSG on IDE degradation of insulin in the presence of 10^−3^ M ascorbate. Ascorbate did not alter either of the curves, suggesting the effects of GSH and GSSG, unlike nitrosylation, are not reversible with the relatively low reducing power of ascorbate. Similar results were obtained with a purified form of IDE (data not shown).

**Figure 8:**
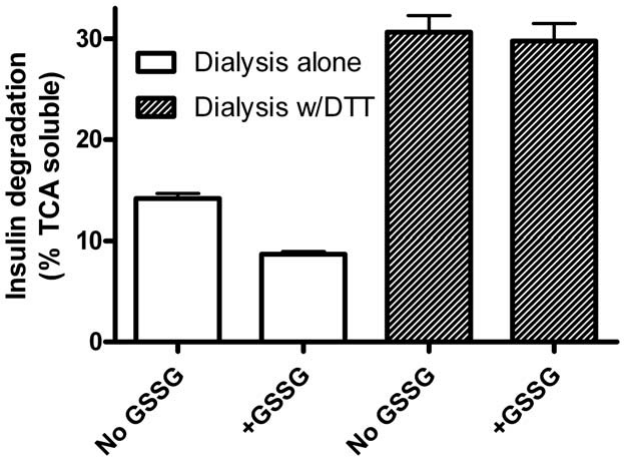
Inhibition of IDE by GSSG is reversible with DTT. IDE was incubated without or with GSSG (10^−4^ M) and then dialyzed in the absence (open bars) or presence (shaded bars) of DTT (10^−2^ M) before assay of insulin degrading activity. GSSG inhibits IDE, while DTT reverses the effect and reveals latent degrading activity. Mean ± SEM of three replicates. The graph is representative of 4 independent experiments with varying levels of insulin degradation (TCA solubility).

If GSSG inhibits IDE by glutathiolation, it should be reversible with a strong enough reducing agent. We therefore incubated IDE without and with 1 mM GSSG, dialyzed the preparations against Tris buffer without and with 1 mM DTT, and assayed for insulin degradation. The final assay did not contain sufficient DTT to reduce the disulfide bonds of insulin (data not shown). The data in Figure 8 show that inhibition by GSSG is reversible with DTT. In fact, DTT treatment increased insulin degrading activity in both the GSSG treated and control preparations. This latent activity suggests the original preparation had some IDE that was already adducted or otherwise inhibited by oxidation consistent with our finding of a low level of glutathionation (Figure 5).

Insulin has three disulfide bonds, two of which join the A-Chain and B-Chain of insulin together, and sufficiently high concentrations of GSH, such as those used in the previous assays, can reduce disulfide bonds. We next examined whether the observed increase in insulin degradation by GSH was due to an increase in enzyme activity or an increase in substrate solubility via a direct effect of GSH on partially-degraded substrates. To accomplish this, two known substrates of IDE were used, insulin and Aβ. Aβ does not contain a disulfide bond, thus this compound would not be expected to exhibit alterations in solubility as a result of the reducing capacity of GSH. In addition, we heat inactivated the enzyme at the end of the incubation, and added 10^−3^ M GSH for a further 15 minutes. Any effect of GSH on substrate solubility at this point would be due to a direct effect on the hormone, and not on IDE activity.

**Figure 9:**
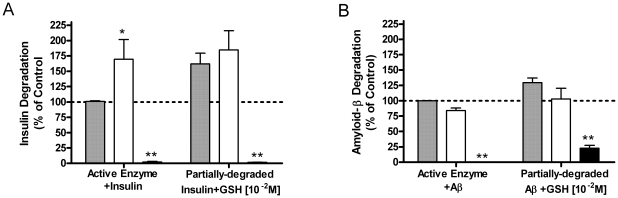
Direct effect of GSH on the partially-degraded ^125^I-insulin and ^125^I-amyloid-β. Substrates were incubated with IDE in the presence of GSH or GSSG [10^−2^ M]; left side of each graph. Alternatively, IDE was incubated with substrate and GSH or GSSG, then IDE was heat-inactivated. An additional 10^−2^ M GSH was added after enzyme inactivation and incubated for another 15 min before TCA precipitation (right side of each graph). Gray bars are Untreated Enzyme; white bars are Enzyme + GSH; black bars are Enzyme + GSSG. (A) Insulin degradation. (B) Amyloid β degradation. Background TCA solubility in the absence of enzyme was subtracted. Treatment with GSH after heat inactivation of the enzyme increases TCA solubility of insulin products by breaking disulfides. Amyloid-β, not having any disulfide bonds, is unaffected either before or after enzyme inactivation. Mean ± SEM of three independent experiments. *P<0.05, **P<0.01 compared to control.

As shown above, GSH appears to increase insulin degradation, while GSSG inhibits it (Figure 9A, left). However, Aβ degradation was unaffected by GSH, while GSSG inhibited (Figure 9B, left), suggesting that GSSG acts on IDE, while GSH only affects the disulfide-containing insulin molecule. When IDE is heat-inactivated at the end of the incubation, and additional GSH added, insulin solubility increases to the level of degradation seen when GSH is included at the start (Figure 9A, right). The addition of GSH to Aβ after IDE inactivation had little effect (Figure 9B, right). In the absence of enzyme, GSH did not significantly increase insulin solubility (data not shown). Thus, GSH is not acting on IDE itself to increase insulin degradation, but instead, is reducing the disulfide bonds of partially-degraded insulin. The insulin disulfide bond reduction effectively increases the TCA solubility of the substrate, but only after partial degradation of insulin has occurred.

**Figure 10:**
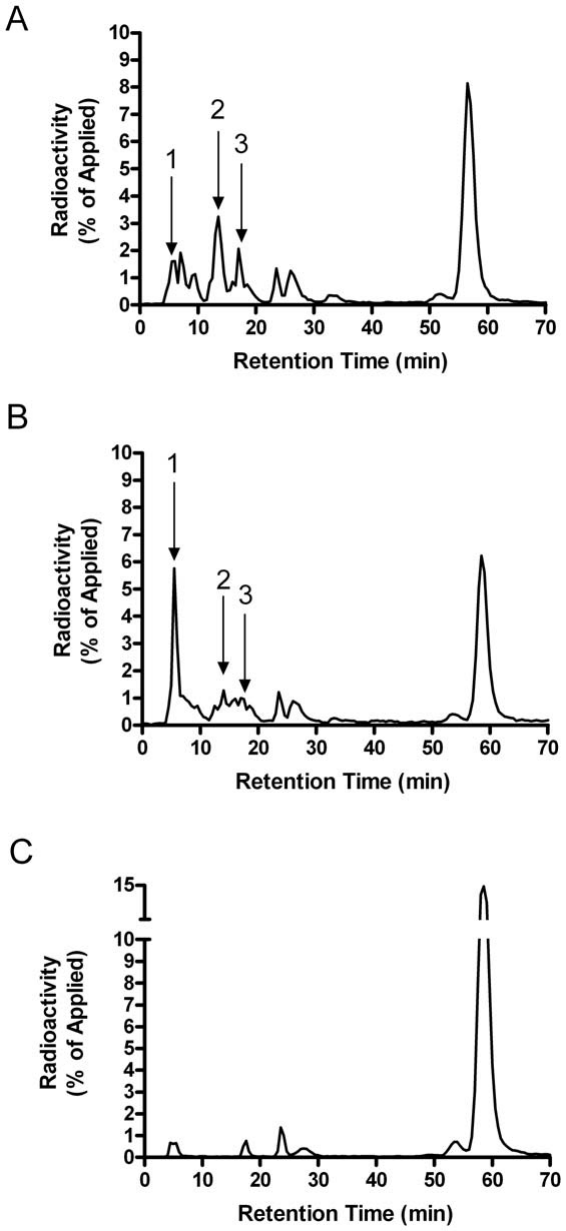
HPLC analysis of insulin degradation products. Insulin degradation products were qualitatively measured after incubation with (A) partially-purified IDE, (B) partially-purified IDE + GSH [10^−2^ M], and (C) partially-purified IDE + GSSG [10^−2^ M]. The peaks that changed in size with GSH treatment are identified with arrows (1, 2, and 3). GSH shifted the product pattern by decreasing peaks 2 and 3, and increasing peak 1. GSSG inhibits IDE and reduced all products.

In order to confirm that GSH was reducing the disulfide bonds of partially-degraded insulin, we examined the insulin-degradation products by HPLC analysis. Figure 10A shows a representative elution profile of ^125^I-insulin and fragments after incubation with partially-purified IDE. Figure 10B shows the insulin elution profile of IDE treated with GSH [10^−2^ M]. GSH treatment resulted in a dramatic increase in the formation of a prominent early insulin product peak (Figure 10A and 10B, peak 1). Previous studies have identified this prominent early peak to contain partially-degraded, trichloroacetic acid soluble, A-chain fragments [26]. There was also a substantial decrease in the production of two later-eluting product peaks (Figure 10A and 10B, peaks 2 and 3). This shift in products is likely to result from the breaking of a disulfide bond, generating smaller less hydrophobic peptides. Treatment of the partially-purified enzyme with 10 mM GSSG significantly inhibited insulin degradation (Figure 10C).

## Discussion

IDE has been linked to T2DM and AD, thus control of its function is of interest regarding the etiology of these diseases. A number of compounds have been reported to alter IDE activity, including fatty acids, ATP, hydrogen peroxide, and NO [11], [12], [34], [35], [36]. Given the thiol sensitivity of IDE, hydrogen peroxide and NO likely react with one or more of the cysteines previously identified to have an effect on activity, namely C178, C789, C812, C819, or C966 [10], [37]. Unlike alkylating reagents such as N-ethylmaleimide, the reaction of these compounds with cysteine residues is reversible. This provides the cell with a potential mechanism to regulate the activity of IDE, depending on the redox state of the cell. But the redox control of IDE may be complex as cysteine C178 has been suggested to provide protection from inactivation by preventing nitrosylation of C110 [37]. However, this effect was seen in a mutated form of IDE with limited cysteines, and whether it is physiologically relevant is uncertain as C110 is not nitrosylated in wild type IDE containing all 13 cysteines. The results presented here add to that complexity and are significant for several reasons.

First, our results confirm previous work showing that NO inhibits insulin degradation, and can alter proteasome activity [11], [12]. The fact that NO inhibited the proteasome only when IDE was present (Figures 3 and 4), provides additional evidence to our previous work [25], [27], [28], [34] that IDE can interact with the proteasome and alter its activity. This is consistent with the known activity of insulin to decrease cellular protein degradation, and suggests IDE may act as an intracellular mediator for insulin action.

Second, we have shown that oxidized glutathione (GSSG) can inhibit IDE, by reacting with one or more of its cysteines. Thus, not only can reactive oxygen species (ROS) such as hydrogen peroxide inhibit IDE directly [35], but increased levels of cellular GSSG, which results from increased ROS, can decrease IDE activity. Although glutathionylation is a reversible form of post-translational modification, it is chemically very different from nitrosylation. The formation of either type of modification does not occur by direct addition of a NO group or GSH to protein cysteines. It is necessary for the cysteine to be in the appropriately reduced state. In order for nitrosylation to occur, the protein must lose *one* electron per sulphur atom and it must lose *two* electrons for glutathionylation, which means that less reducing power is required for the reversal of cysteine nitrosylation [38]. Thus, how IDE is modified will determine how readily the inhibition can be reversed.

Third, while GSSG inhibits IDE, reduced glutathione (GSH) appears to increase insulin degradation. This was not due to an effect on IDE, but rather on the ability of GSH to reduce the disulfides in partially degraded insulin as confirmed by HPLC analysis of the products (Figure 10). Intact insulin is not affected by GSH. Thus, the redox state of the cell can affect the rate at which insulin is degraded to small peptides. The effects of both compounds are seen in the mM range, consistent with the typical cellular concentration of glutathione. Normally the ratio of GSH to GSSG is around 10, which would strongly favor insulin breakdown. However, because GSH and GSSG have reciprocal action in the same concentration range (Fig. 7), a shift toward the oxidized state would act to slow insulin degradation; i.e. less GSH to break the disulfide and more GSSG to inhibit IDE. The effect of a more oxidizing environment (including increased GSSG concentrations) would be more evident on lowering Aβ degrading activity, as GSH has no effect to increase its degradation (Figure 9). These data, along with the previous studies on ROS and RNS, suggest IDE activity is subject to fine control by ambient conditions of the cell. Indeed, our results treating the partially purified enzyme with DTT (Figure 8) and the low level of endogenous glutathionylation (Figure 5) suggest that IDE is already partly inhibited upon isolation, although it cannot be ruled out that this is an artifact of the purification procedure.

Finally, the effect of GSH is also of interest in historical terms. In the late 1970's and early 1980's there was some debate whether IDE or glutathione insulin transhydrogenase (GIT, now identified as protein disulfide isomerase, PDI) acted first on insulin [39]. The current consensus is that IDE acts first, and our results indicate that PDI is not required for the action of GSH, after initial cleavage by IDE. The non-enzymatic action of GSH may explain why early studies suggested a role for PDI, when the susceptibility of minimally degraded insulin was unknown. This action of GSH may also explain why the insulin cleavage products that accumulate in cells are different than those generated by IDE *in vitro* or extracellularly [40], [41]. The HPLC pattern of extracellular ^125^I-iodoinsulin degradation products is the same as that generated by purified preparations IDE, with numerous peaks evident. However, insulin-sized intracellular products are more limited in number, presumably due to their rapid breakdown by GSH.

In conclusion the results presented here indicate that insulin degradation by IDE can be regulated in both positive and negative ways. We have shown for the first time that IDE can be glutathionylated by oxidized glutathione. Both nitrosylated and glutathionylated, forms of IDE are inhibited with respect to insulin degradation. These modifications are reversible, but require less reducing power for nitrosylation than for glutathionylation. Insulin degradation can also be accelerated by direct interaction of GSH with insulin, but only after partial cleavage by IDE, which presumably allows for distortion of the insulin molecule and exposure of the disulfide bonds. These findings suggest that cellular conditions that contribute to NO availability or a more oxidizing environment may decrease the degradation of insulin or amyloid β and contribute to the etiology of T2DM or Alzheimer's disease, respectively.
